# Health insurance is important in improving maternal health service utilization in Tanzania—analysis of the 2011/2012 Tanzania HIV/AIDS and malaria indicator survey

**DOI:** 10.1186/s12913-018-2924-1

**Published:** 2018-02-13

**Authors:** Stephen M. Kibusi, Bruno Fokas Sunguya, Eunice Kimunai, Courtney S. Hines

**Affiliations:** 1 School of Nursing and Public Health, College of Health Sciences, University of Dodoma, Dodoma, Tanzania; 2 Department of Community Health, School of Public Health and Social Sciences, Muhimbili University of Health and Allied Sciences, Dar es Saalam, Tanzania

**Keywords:** Health insurance, Antenatal care, Skilled birth delivery, Maternal health services

## Abstract

**Background:**

Maternal mortality rates vary significantly from region to region. Interventions such as early and planned antenatal care attendance and facility delivery with skilled health workers can potentially reduce maternal mortality rates. Several factors can be attributed to antenatal care attendance, or lack thereof, including the cost of health care services. The aim of this study was to examine the role of health insurance coverage in utilization of maternal health services in Tanzania.

**Methods:**

Secondary data analysis was conducted on the nationally representative sample of men and women aged 15–49 years using the 2011/12 Tanzania HIV and Malaria Indicator Survey. It included 4513 women who had one or more live births within three years before the survey. The independent variable was health insurance coverage. Outcome variables included proper timing of the first antenatal care visit, completing the recommended number of antenatal care (ANC) visits, and giving birth under skilled worker. Data were analyzed both descriptively and using regression analyses to examine independent association of health insurance and maternal health services.

**Results:**

Of 4513 women, only 281 (6.2%) had health insurance. Among all participants, only 16.9%, 7.1%, and 56.5%, respectively, made their first ANC visit as per recommendation, completed the recommended number of ANC visits, and had skilled birth assistance at delivery. A higher proportion of women with health insurance had a proper timing of 1st ANC attendance compared to their counterparts (27.0% vs. 16.0%, *p* < 0.001). Similar trend was for skilled birth attendance (77.6% vs. 55.1%, *p* < 0.001). After adjusting for other confounders and covariates, having health insurance was associated with proper timing of 1st ANC attendance (AOR = 1.89, *p* < 0.001) and skilled birth attendance (AOR = 2.01, *p* < 0.01).

**Conclusions:**

Health insurance coverage and maternal health services were low in this nationally representative sample in Tanzania. Women covered by health insurance were more likely to have proper timing of the first antenatal visit and receive skilled birth assistance at delivery. To improve maternal health, health insurance alone is however not enough. It is important to improve other pillars of health system to attain and sustain better maternal health in Tanzania and areas with similar contexts.

## Background

Maternal mortality has decreased significantly in the past two decades [1, 2]. Globally, the rate of annual decline of maternal deaths increased from 0.3% from 1990 to 2003 to 2.7% between 2003 and 2013 [1]. This decline however, varies with regions. While some regions show fast decline in maternal mortality ratios (MMR), others are stagnant and the rest display upward trends. In sub-Saharan Africa in particular, MMR have been increasing despite the efforts of various programs and initiatives. To this end, Tanzania is no different [3]. In Tanzania, MMR per 100,000 live births was 578 in 2015, 454 in 2010, and 432 in 2012 [3–5]. The most recent data in Tanzania shows further increase in MMR to 556 per 100,000 live births [4]. The rate of annual decline in maternal mortality in the last decade was only 1.1% compared to 4.7% in the previous decade [1]. Such poor trend was behind the failure to reach the Millennium Development Goal five (MDG5) that expired in 2015 [6].

High MMR in Tanzania and slow decline thereof is caused by a myriad of underlying factors. Like other countries in the region, Tanzania faces poor health systems with few qualified health workers, inequality in health facilities distribution, high out-of-pocket health expenditures, food insecurity and poor nutrition status in some regions, poverty, low maternal education levels, and diseases, including HIV [6–8]. As a result, a high proportion of mothers choose to deliver at home, unassisted by skilled health workers, even if they attend antenatal clinics (ANC) [3].

Early and planned ANC attendance and facility delivery with skilled health workers can potentially reduce the risks of immediate causes of MMR [9, 10]. These causes include: pregnancy induced hypertension, malaria infection during pregnancy, vaccine-preventable diseases such as rubella and tetanus, high risk pregnancies such as multipara and multiple gestation, and prenatal, natal, and post natal hemorrhage [11]. ANC attendance and other community based interventions for maternal health were associated with facility delivery in some countries [12]. In Tanzania as high as 96.5% of women attend ANC at least once; however, a fraction of them attend the four required ANC visits and only 42.6% of births are attended by skilled personnel [3].

A myriad of factors contribute to inadequate ANC attendance and unassisted delivery in Tanzania and countries with similar characteristics. Such factors include long distance to health facilities, low economic status, low levels of maternal education and poor knowledge of danger signs, lack of skilled workers and facilities, and high financial burden to families of expectant mothers [13–15]. In Tanzania, the government subsidizes maternal health services and user-fee exemption is provided in public health facilities [16–18]. However, pregnant women encounter a number of hidden expenses when pursuing health services [19]. Such expenses may arise from the long distance travelled to seek care, lack of essential medicine and equipment in public facilities necessitating them to buy in private drug stores, unofficial payment to the health workers, and time spent due to the heavy burden of patients in facilities with inadequate numbers of health workers [13, 14, 16, 19]. Such high out-of-pocket (OOP) expenditures introduce barriers to accessing maternal health services in Tanzania [19].

Health insurance is an effective mechanism to mitigate OOP expenditures [20]. It reduces catastrophic payments, and can improve health seeking and utilization behaviors [21]. It is also associated with ANC attendance and may increase the number of deliveries attended by skilled health workers and the number of deliveries that take place in a health facility [7]. Under such context, health insurance has a role in improving MMR and other related health problems. However, only a fraction of the population in Tanzania has access to health insurance [3, 5, 22]. Health insurance coverage is also not equally distributed along the socio- economic and employment lines [22]. Various forms of health insurance exist for individuals in informal sectors, including community health funds, but distribution of coverage varies with areas of implementation [22].

Evidence is available on the association between health insurance and maternal service utilization in developed countries [7, 23–25]. However, such evidence is not available in developing countries, especially where insurance coverage is low; Tanzania is no exception. This study aims to examine health insurance coverage and its association with maternal health services utilization in Tanzania.

## Methods

### Study design

This cross-sectional study was designed to utilize secondary data originated from the nationally representative sample of men and women aged 15–49 years in the 2011/12 Tanzania HIV/AIDS and Malaria Indicator Survey (THMIS). Data were collected between December 2011 and May 2012. The National Bureau of Statistics (NBS) developed the sampling frame used for the survey to cover all 30 regions of Tanzania. It excluded nomadic and institutional populations such as persons in hotels, barracks, and prisons.

### Sampling procedures

The sample was selected in two stages. The first stage involved the selection of 585 clusters (sample units) consisting of enumeration areas (EAs). On the mainland, 30 clusters were selected from Dar es Salaam and 20 were selected from each of the other 24 regions. In Zanzibar, 15 sample points were selected from each of the five regions.

The second stage of selection involved the systematic sampling of households. A household listing operation was undertaken in all of the selected areas prior to the fieldwork. From these lists, households to be included in the survey were selected. Approximately 18 households were selected from each sample point for a total sample size of 10,496 households. Weighting factors were added to the data file so that the results would be proportional at the national level. Eligibility criteria included women and men ages 15–49 who were either permanent residents of the selected households or visitors who stayed in the household the night before the survey were.

### Data source

Data analyzed in this study came from the Individual Questionnaire of the 2011/2012 THMIS dataset. It included a total of 19,319 men and women. Out of them, a total of 8352 men were excluded to get the study population of women who had a live birth within three years prior to the survey. Out of the 10,967 women remained, only 4627 women had one or more live births in the past three years before the survey were selected. A total of 114 women were excluded for missing data on important variables. They include 14 women who were missing information on insurance coverage and 100 women who were missing information on the timing of the first antenatal visit. Finally, 4513 women who had one or more live births within three years before the survey were included for analysis. The data set for this survey is available upon request from Measures, DHS program. The URL for this specific dataset is https://www.dhsprogram.com/data/dataset_admin/login_main.cfm;jsessionid=4E33501BD1C8593FA55A278F027BFD44.cfusion?CFID=12989464&CFTOKEN=c9b926a975283d4-05E5988A-9609-2AFA-E0772649E1FDACED

### Variables and measurements

Variables were identified from the 2011–12 THMIS individual questionnaires and selected based on previous literature. The outcome variable of interest was maternal health services utilization. Three items from the THMIS individual questionnaire were used to measure it. They include: timing of the first antenatal care visit, completing the recommended number of ANC visits, and giving birth at a health facility under skilled care. Timing of the first ANC visit is recommended during the first trimester. Four spaced visits are the minimum number of recommended focused ANC visits. Lastly, a woman is considered as having received skilled birth assistance at delivery if she gave birth in a health facility, under the care of a trained health worker with the necessary skills for assisting with birth [3].

The main independent variable in this study was health insurance coverage. Participants were asked if they have any type of health insurance eliciting a dichotomized response of “Yes” or “No.” Other covariates and confounders in this analysis included socioeconomic and demographic variables. Demographic variables included age clustered in five-year age groups, rural or urban area of residence, education level, marital status, occupation, and wealth status. Number of births in the past five years before the survey was dichotomized into “one child” or “2 or more births.” Tanzania’s regions were regrouped into nine geographical zones, including Eastern, Western, Southern, Southern High, South West Highlands, Central, Northern, Lake and the Zanzibar zone.

We used weighted wealth index to assess economic status. It was constructed using ownership of household assets ranging from a television to a bicycle or car; dwelling characteristics, such as source of drinking water, sanitation facilities, and type of flooring material; and household’s food availability or consumption. Three steps were undertaken; first, a subset of indicators common to urban and rural areas was used to create wealth scores for households in both areas. Second, separate factor scores were produced for households in urban and rural areas using area-specific indicators using principle component analysis. Third, the separate area-specific factor scores were combined to produce a nationally applicable combined wealth index by adjusting area-specific scores through a regression on the common factor scores. Wealth quintiles (from lowest to highest) were formed by dividing the generated wealth index into the five rankings—the wealth index quintiles.

### Data analyses

Analysis was conducted using both descriptive and regression methods. First, descriptive statistics on the distribution of participants’ socio-demographic characteristics by health insurance coverage and utilization of maternal health services were collected. For maternal services utilization, timing of the first antenatal visit, completing the recommended number of antenatal visits, and giving birth under skilled labor attendance were analyzed. The level of significance was set at *P* < 0.05 (2-tailed) for all the analyses.

A multiple logistic regression was conducted to examine independent associations between health insurance coverage with the three items used to measure maternal health services utilization. The generated adjusted odds ratios (AOR) were estimated to assess the strength of the associations and used the 95% confidence intervals (CIs) for significance testing. All the covariates were entered simultaneously into the multiple regression models. Analyses were performed using SPSS version 16. Sample weighting was applied to allow for adjustments for the cluster sampling design and sampling probabilities across clusters and strata.

## Results

### Health insurance and related characteristics

Data of 4513 participants was available for analysis. Health insurance coverage was low. Of all participants, only 281 (6.2%) reported to have health insurance. Among those insured 167 (59.5%) were covered under community health insurance schemes, 95 (33.8%) covered under employment based health insurance schemes and the remaining 4.5% and 1.8% were covered under social security funds and other types of insurance schemes respectively.

Table 1 shows the results of descriptive characteristics stratified by health insurance coverage. Health insurance coverage varied with education level, occupation, marital status, place of residence, and wealth index. For example, it increased from non-educated women to secondary and above level (2.8% to 13.2%, *p* < 0.001). It also increased from low wealth index (3.6% to 12.7%, *p* < 0.001). Women residing in rural areas had lower rates compared to their counterparts in urban areas (5.8% vs. 8.5%, *p* < 0.01). Unemployed individuals had lower coverage compared to their employed counterparts (5.4% vs. 23.2%, *p* < 0.001). Health insurance coverage was high among women at the age groups 25–39 years.

**Table 1:** The distribution of participants by socio-demographic characteristics and health insurance coverage (*N* = 4513)

Variables	Health insurance coverage				*P*-value
	Not covered		Covered		
	n	%	n	%	
Education:					
No education	984	97.2	28	2.8	***
Primary	2730	94.0	174	6.0	
Secondary+	518	86.8	79	13.2	
Age groups:					
15–19	381	96.2	15	3.8	
20–24	1034	94.3	63	5.7	
25–29	1074	92.8	83	7.2	
30–34	767	93.7	52	6.3	
35–39	626	94.5	49	7.3	
40–44	275	96.2	16	5.5	
45–49	75	93.2	3	3.8	
Occupation:					
Unemployed	541	94.6	31	5.4	***
Self employed	2958	94.4	175	5.6	
Manual labor	594	94.7	33	5.3	
Employed	139	76.8	42	23.2	
Marital Status:					
Never married	290	92.1	25	7.9	**
Married/living together	3533	93.6	243	6.4	
Divorced/Separated	409	96.9	13	3.1	
Area of residence:					
Urban	666	91.5	62	8.5	**
Rural	3566	94.2	219	5.8	
Wealth:					
Poorest	918	96.4	34	3.6	***
Poorer	921	94.9	50	5.1	
Middle	907	95.0	48	5.0	
Richer	860	93.7	58	6.3	
Richest	626	87.3	91	12.7	

Here *, ** and *** indicate *p* < 0.05, *p* < 0.01 and *p* < 0.001 respectively

### Maternal health service utilization

Table 2 shows the characteristics of 4513 study participants stratified by their utilization of maternal health services. Utilization of maternal health services was categorized into timing of first ANC visit, completion of the recommended ANC visits, and attended by skilled health worker during labor.

**Table 2:** Distribution of participants by factors affecting use of maternal and child health services during pregnancy and birth in Tanzania (*N* = 4513)

Variable	Timing of the 1st ANC Visit					Completed recommended number of ANC visits					Birth attended by a skilled worker				
	Yes		No		*P*-value	Yes		No		*P*-value	Yes		No		*P*-value
	N	%	N	%		N	%	N	%		N	%	N	%	
Health insurance coverage:															
Yes	76	27.0	205	73.0	***	19	6.8	262	93.2		218	77.6	63	22.4	***
No	686	16.2	3546	83.8		303	7.2	3929	92.8		2332	55.1	1900	44.9	
Age group:															
15–19	62	15.7	334	84.3	**	2	0.5	394	99.5	**	262	66.2	134	33.8	**
20–24	229	20.9	868	79.1		77	7.0	1020	93.0		627	57.2	470	42.8	
25–29	197	17.0	960	83.0		103	8.9	1054	91.1		657	56.8	500	43.2	
30–34	117	14.3	702	85.7		71	8.7	748	91.3		442	54.0	377	46.0	
35–39	108	16.0	567	84.0		56	8.3	619	91.7		376	55.7	299	44.3	
40–44	40	13.7	251	86.3		11	3.8	280	96.2		144	49.5	147	50.5	
45–49	9	11.5	69	88.5		2	2.6	76	97.4		42	53.8	36	46.2	
Area of Residence:															
Rural	149	20.5	579	79.5	***	16	2.2	712	97.8	***	627	86.1	101	13.9	***
Urban	613	16.2	3751	83.1		306	8.1	3479	91.9		1923	50.8	1862	49.2	
Education level:															
No education	127	12.5	885	87.5	***	112	11.1	900	88.9	***	400	39.5	612	60.5	***
Primary incomplete	125	18.8	541	81.2		59	8.9	607	91.1		332	49.8	334	50.2	
Primary Complete	406	18.1	1832	81.9		131	5.9	2107	94.1		1359	60.7	879	39.3	
Secondary or higher	104	17.4	493	82.6		20	3.4	577	96.6		459	76.9	138	23.1	
Wealth:															
Poorest	137	14.4	815	85.6	***	81	8.5	871	91.5	***	372	39.1	580	60.9	***
Poorer	141	14.5	830	85.5		96	9.9	875	90.1		458	47.2	513	52.8	
Middle	147	15.4	808	84.6		78	8.2	877	91.8		486	50.9	469	49.1	
Richer	169	18.4	749	81.6		50	5.4	868	94.6		612	66.7	306	33.3	
Richest	168	23.4	549	76.6		17	2.4	700	97.6		622	86.8	95	13.2	
Marital status:															
Single	58	18.4	257	81.6	0.524	3	1.0	312	99.0	***	238	75.6	77	24.4	***
Married	627	16.6	3149	83.4		297	7.9	3479	92.1		2087	55.3	1689	44.7	
Separated/Widowed	77	18.2	345	81.8		22	5.2	400	94.8		225	53.3	197	46.7	
Occupation:															
Unemployed	108	18.9	462	81.1	***	36	6.3	534	93.7	***	380	66.7	190	33.3	***
Self employed	87	22.8	295	77.2		24	6.3	358	93.7		198	51.8	184	48.2	
Manual laborer	128	20.4	499	79.6		18	2.9	609	97.1		467	74.5	160	25.5	
Employed	438	14.9	2494	85.1		244	8.3	2688	91.7		1504	51.3	1428	48.7	
Number of births in last 5 yrs.:															
1	444	20.2	1758	79.8	***	0	0.0	2202	100.0	***	1488	67.6	714	32.4	***
2 or more	318	13.8	1993	86.2		322	13.9	1989	86.1		1062	46.0	1249	54.0	
Zone:															
Eastern	127	31.1	282	68.9	***	11	2.7	398	97.3	***	337	82.4	72	17.6	***
Western	55	13.6	349	86.4		34	8.4	370	91.6		220	54.5	184	45.5	
Southern	64	32.2	135	67.8		7	3.5	192	96.5		116	58.3	83	41.7	
Southern Highlands	91	25.0	273	75.0		9	2.5	355	97.5		327	89.8	37	10.2	
South West highlands	66	13.4	425	86.6		31	6.3	460	3.7		240	48.9	251	51.1	
Central	53	11.3	414	88.7		41	8.8	426	91.2		238	51.0	229	49.0	
Northern	64	19.2	270	80.8		8	2.4	326	97.6		183	54.8	151	45.2	
Lake	148	11.7	1120	88.3		131	10.3	1137	89.7		589	46.5	679	53.5	
Zanzibar	94	16.3	483	83.7		50	8.7	527	91.3		300	52.0	277	48.0	

Here *, ** and *** indicate *p* < 0.05, *p* < 0.01 and *p* < 0.001 respectively

In this study, timing of the first ANC visit was also low. Only 762 (16.9%) of participants reported proper timing. A higher proportion of those with health insurance had proper timing of ANC compared to their counterparts (27.0% vs. 16.2%, *p* < 0.001). Timing of the first ANC visit differed across various socio-demographic characteristics. For example, it was higher among women in the age group 20–24 (20.9%), residing in rural areas (20.5%), and women with at least primary education or higher education (primary incomplete 18.8%, primary complete 18.1% and secondary or higher education 17.4%) compared to those in other age groups (χ2 test *p* < 0.01), residing in urban areas or with no formal education (χ2 test *p* < 0.001). Also, timing of the first antenatal care visit increased proportionally with the wealth index. For example, 23.4% and 18.4% of those in the richest and richer quintile, respectively, had recommended timing of first visit compared with only 14.4% of the poorest group quintile (χ2 test *p* < 0.001). Only 14.9% of the employed mothers had proper timing of the first ANC visit compared to 22.8% among those who were self-employed (χ2 test *p* < 0.001). There were proportionally significant variations across the nine survey zones with the Eastern zone showing the highest proportion of women timing their first antenatal care visit as recommended (31.1%) compared to only 11.7% and 11.3% the Lake and the Central zones respectively (χ2 test *p* < 0.001).

A few women had completed the recommended ANC visits (Table 2). Only 322 (7.1%) completed four recommended ANC visits. There was no difference between those with or without health insurance under such context (6.8% vs. 7.2%, *p* = 0.460). Younger women were less likely to complete all four recommended ANC visits compared to other age groups (χ2 test *p* < 0.01). This was also true for the urban-rural residency (2.2% vs. 8.1%, *p* < 0.001). Those with low education had higher completion rates compared to the higher education cluster (χ2 test *p* < 0.001). Similarly, women of the richest quintile had the lowest completion rate of ANC visits compared to poorest quintile (2.4% vs. 9.9%, *p* < 0.001). Completion rate was also lower among single women compared to their married counterparts (χ2 test *p* < 0.001); and those residing in the Eastern zones (χ2 test *p* < 0.001).

Lastly, majority of the selected population (56.5%) reported to have delivered at health facilities under skilled attendants. A higher proportion of them had health insurance compared to their counterparts (77.6% vs. 55.1%, *p* < 0.001). A high number [1963 (43.5%)] of women did not have their birth attended by a skilled health worker. More young women gave birth in healthcare facilities under skilled health workers compared to older groups (χ2 test *p* < 0.01). Counter intuitively, a significantly high proportion of women residing in rural areas gave birth under skilled health workers than those in urban areas (86.1% vs. 50.8% *p* < 0.001). About 76.9% of women with secondary or higher education gave birth under skilled attendance compared with only 39.5% of those with no education (χ2 test *p* < 0.001). Women of the richer quintile category received skilled attendance at birth in higher proportion compared to those of the poorest wealth quintile (86.0% vs 39.1%, *p* < 0.001). Contrary to the results of ANC completion rates, 75.6% of single women gave birth under skilled attendance, while only 55.3% of the married women gave birth under skilled attendants (χ2 test *p* < 0.001). Occupational status, parity and the geographical zone also showed significance differences with skilled birth in this survey (Table 2).

### Independent associations between health insurance and maternal services utilization

Table 3 shows the results of a multivariate logistic regression analysis for the association between health insurance coverage and utilization of maternal health services. After controlling for other factors, health insurance coverage was associated with increased odds of timing the first antenatal visit as recommended (OR 1.86, *p* < 0.001). Women with health insurance were also more likely to deliver at a health facility under a skilled attendant compared to their counterparts (AOR 2.01 *p* < 0.001). There was no significant association between health insurance coverage and completing the recommended number of antenatal visits (AOR 1.33, 95% CI 0.782–2.267).

**Table 3:** Adjusted Odds Ratios (AOR) for the association between health insurance coverage and maternal health service utilization among women of reproductive age in Tanzania (*N* = 4513)

Variable	Timing of the first Antenatal visit			Completing recommended number of antenatal visits			Birth attended by a skilled health worker		
	AOR	95% CI	*P* value	AOR	95% CI	*P* value	AOR	95% CI	*P* value
Health insurance coverage:									
No	1			1			1		
Yes	1.855	1.375–2.503	***	1.331	0.782–2.267		2.012	1.458–2.775	***
Age groups:									
15–19	1			1			1		
20–24	1.465	1.060–2.026	**	5.367	1.280–22.51	**	0.041	0.754–0.576	*
25–29	1.055	0.751–1.483		5.786	1.385–24.17	**	0.023	0.723–0.546	*
30–34	0.836	0.581–1.202		5.874	1.396–24.71	**	0.008	0.677–0.506	**
35–39	0.949	0.657–1.370		5.883	1.390–24.91	**	0.106	0.783–0.582	
40–44	0.795	0.506–1.250		2.911	0.621–13.65		0.006	0.614–0.432	**
45–49	0.673	0.313–1.450		2.254	0.298–17.06		0.633	0.877–0.511	
Area of Residence:									
Rural	1			1			1		
Urban	1.255	1.054–1.345	**	1.454	0.775–2.726		0.511	0.388–0.673	***
Marital Status:									
Single	1			1			1		
Married	1.202	0.861–1.679		1.625	0.485–5.436		0.899	0.656–1.231	
Separated/widowed	1.327	0.880–2.002		1.325	0.367–4.782		0.732	0.504–1.063	
Zones:									
Eastern	1			1			1		
Western	0.391	0.268–0.572	***	1.086	0.516–2.287		0.654	0.457–0.937	*
Southern	1.118	0.756–1.653		1.355	0.477–3.847		0.626	0.412–0.951	*
Southern Highlands	0.765	0.543–1.079	***	0.526	0.204–1.352		3.669	2.325–5.790	***
Southern West Highland	0.381	0.266–0.546	***	0.853	0.402–1.809		0.475	0.335–0.673	***
Central	0.298	0.204–0.436	**	1.432	0.688–2.983		0.499	0.352–0.709	***
Northern	0.568	0.398–0.809	***	0.483	0.184–1.265		0.360	0.248–0.522	***
Lake	0.323	0.238–0.438	***	1.414	0.719–2.782		0.448	0.327–0.615	***
Zanzibar	0.512	0.363–0.722	***	1.641	0.784–3.434		0.210	0.146–0.303	***
Births in the past 5 yrs.:									
One	1			1			1		
Two or more	1.277	1.071–1.523	**	1.681	0.000		0.589	0.510–0.679	***
Educational level:									
No education	1			1			1		
Primary incomplete	1.415	1.071–1.869	**	0.974	0.681–1.393		1.209	0.974–1.500	
Primary Complete	1.212	0.962–1.528		0.769	0.575–1.029		1.335	1.126–1.583	**
Secondary or higher	0.817	0.581–1.150		0.658	0.365–1.186		2.102	1.564–2.824	***
Wealth:									
Poorest	1			1			1		
Poorer	0.988	0.79–1.286		1.200	0.859–1.676		1.326	1.092–1.609	**
Middle	1.082	0.830–1.411		0.989	0.696–1.406		1.463	1.200–1.783	***
Richer	1.228	0.931–1.621		0.829	0.544–1.262		2.369	1.898–2.958	***
Richest	1.605	1.126–2.288	**	0.686	0.344–1.369		4.527	3.239–6.326	***
Occupation:									
Unemployed	1			1			1		
Self employed	1.104	0.828–1.473		0.930	0.587–1.473		0.760	0.591–0.978	*
Manual laborer	1.191	0.884–1.605		0.547	0.296–1.009		1.306	0.978–1.743	
Employed	1.254	0.808–1.946		0.508	0.144–1.788		1.289	0.784–2.120	

Here *, ** and *** indicate *p* < 0.05, *p* < 0.01 and *p* < 0.001 respectively

Other factors associated with timing the first antenatal visit as recommended were being in the 20–24 age group compared to the youngest age group (AOR 1.47 *p* < 0.01) and residing in an urban compared to rural area (AOR 1.26 *p* < 0.01). In the ANC completion rate model, age was the only factor associated with completing the recommended number of antenatal visits. Compared with young women in the 15–19 years, being older increased the odds of completing the recommended number of antenatal visits (*p* < 0.01).

Table 3 also presents results of a regression analysis in the skilled birth attendants’ model. Counter intuitively; women residing in urban areas were less likely to deliver under skilled workers (AOR 0.511 *p* < 0.001). Being a young mother (ages 15–19) increased the odds of delivering at a health facility compared to those in 20–24 years and above age groups. Residents from the Southern highlands were more likely to deliver under skilled health worker than those from the Eastern zone (AOR 3.67 *p* < 0.001).

## Discussion

Health insurance coverage was low in this study. Only 6.2% of women in this study had health insurance. Maternal health services utilization was also low. For example, only 16.9% of all women in this study had a timing of first ANC initiation as recommended. Moreover, only 7.1% completed four recommended ANC visits and about 43.5% did not deliver their last baby under a skilled attendant. This result is not far from previous studies in Tanzania on skilled birth delivery [14, 15]. Having a health insurance was associated with proper timing of ANC initiation and giving birth under a skilled attendant. Results of this secondary analysis will help shade some light into the challenge ahead in trying to achieve the universal health and further reduce the otherwise persistent maternal mortality rate in Tanzania.

Low health insurance coverage was associated with low rates of maternal services utilization in this study. To counter low rates of maternal health services utilization, Tanzania, like many other countries in sub-Saharan Africa introduced policies to provide free medical care to pregnant women. Removing the financial barriers to antenatal care is thought to help increasing utilization of ANC services [26, 27]. However, lack of equipment, including essential medicine, introduces hidden user fee [14, 19], especially for those with no insurance coverage. These women have to purchase such medicine and equipment in private pharmacies, therefore, increase the rates of out-of-pocket payments. In Tanzania, 73.3% of women with facility delivery reported having made out-of-pocket payments for delivery-related costs, including unofficial provider payments [19]. Like previous studies, health insurance coverage may therefore increase the likelihood of enrolment and utilization of ANC [7, 26].

Like in a previous study [7], our results confirmed that parity, age, education level, wealth index, place of residence influences timing of ANC initiation. Specifically, young women, those who lived in urban areas, with two or more births, and with highest economic status were more likely to adhere to the recommended timing of ANC initiation. Women of high wealth quintile are more likely to also be educated, have knowledge of risk factors for pregnancy, and therefore adhere to the recommended timing of ANC initiation. In this study however, women having incomplete primary education level were more likely to adhere to this recommendation compared to non-educated. Associations with other education levels did not reach statistical significant levels. Also, women with higher parity are more likely to have more knowledge of risk factors and therefore adhere to this recommendation compared with the primigravida [7]. There was a regional variation of ANC initiation in this study. The Eastern zone had the best indicators of ANC initiation. Efforts should be done in other zones by looking at the strategies that have been effective in the Eastern zone.

Health insurance was not associated with completion rates of ANC visits in this study. However, this study shows that, age was an important factor for ANC completion rate. Women who were older had adhered to the recommendation of at least four ANC visits compared to the youngest age group (15–19). This finding is similar to previous study in different settings [7]. In India for example, adolescents had unacceptably low utilization of maternal health services [28]. Similar findings were reported elsewhere [29, 30].

In contrast to other studies [14, 31], our study found that women of urban areas were less likely to deliver under skilled health workers compared to their rural counterparts. Also, similar to our study, wealth index was positively associated with skilled birth delivery [31]. This can be explained by the knowledge that, wealthier women are more likely to be empowered or educated, and therefore are aware of the maternal risk or can access health facilities even under hidden costs [14]. Also, our study found that, younger women were more likely to deliver under skilled health workers. Similar to the ANC initiation, the Eastern zone had highest rate of skilled birth delivery which coincide with another study where focused ANC care was the highest in this region [13].

Results of this study should be carefully discussed owing to the following limitations. First, we only examined women who had a live birth in the past three years to minimize recall period. Therefore, we cannot generalize our findings to all other women. Second, the primary source of the information collected from the research participants was self-report. Such method could have introduced recall bias that could have resulted into underestimation or overestimation of past experiences or events. We minimized this effect through limiting the sample to only those who gave a live birth in the past three years. Third, we were limited on the variables that were available in the DHS questionnaire; thus, we could not explore other factors that could be relevant for this study. To minimize the effect of this limitation, we included as many variables from the questionnaire into our conceptual model. Despite these limitations, this is the first study that examined the role of health insurance in maternal health utilization in Tanzania.

## Conclusions

In conclusion, health insurance coverage was low in Tanzania. Also, maternal services utilization such as recommended timing of ANC initiation, completion of recommended number of ANC visits, and skilled birth delivery was low. Having health insurance was associated with recommended timing the first ANC visit and increases the chances for health facility delivery under skilled health worker. Our results highlight the potential role of health insurance in improving maternal health and therefore address areas of improvement in the newly introduced Sustainable Development Goals number three and five. These study findings also provide guidance for policy makers in low- and middle-income countries on the role of health insurance coverage in utilization of maternal health services. Increase in health insurance coverage alone may not bring about the desirable changes in maternal health. It is also important to address other health system challenges to attain and sustain better maternal health in this settings. Such challenges are on the health system pillars such as poor human resource for health, medical supplies, information technology, and stewardship.

## Abbreviations

ANC: Antenatal Clinic
EAs: Enumeration areas
MDG5: Millennium Development Goal five
MMR: Maternal Mortality Ratio
NBS: National Bureau of Statistics
NIMR: National Institute of Medical Research
OOP: Out-of-pocket
THMIS: Tanzania HIV/AIDS and Malaria Indicator Survey
ZAMREC: Zanzibar Medical Research and Ethics Committee
